# The Role of Ti in the Improvement of Thermoelectric Performance in Nb_0.6−*x*_Ti_*x*_Ta_0.4_FeSb

**DOI:** 10.1021/acs.chemmater.6c00307

**Published:** 2026-06-20

**Authors:** Panagiotis Mangelis, Michal Rybski, Mingming Guo, Panagiotis S. Ioannou, Savvas Hadjipanteli, Panagiotis Koutsogiannis, Ioannis Thanoglou, Andreas Delimitis, Laurent Chaput, Janusz Tobola, Theodora Kyratsi

**Affiliations:** † Department of Mechanical and Manufacturing Engineering, 54557 University of Cyprus, Nicosia 2109, Cyprus; ‡ Faculty of Physics and Applied Computer Science, 49811 AGH University, Krakow 30-059, Poland; § 137665 Université de Lorraine, CNRS, LEMTA, Nancy F-54000, France; ∥ Electron Microscopy Core Facility, 54557 University of Cyprus, Nicosia 2109, Cyprus; ⊥ Department of Physics, 37782 Aristotle University of Thessaloniki, Thessaloniki 54124, Greece

## Abstract

Half-Heusler (HH) materials with the stoichiometry Nb_0.6−*x*_Ti_*x*_Ta_0.4_FeSb (0 ≤ *x* ≤ 0.175) were synthesized by mechanical alloying. The role of Ti doping on electrical and thermal transport properties is studied by both experimental and theoretical approaches. Powder X-ray diffraction, Rietveld refinements, and transmission electron microscopy analyses confirm the HH phases and the incorporation of Ti atoms in the crystal structure. Electrical conductivity and Seebeck coefficient measurements indicate that Ti substitution induces an effective hole doping, significantly increasing σ. However, the increase in hole concentration is accompanied by a moderate decrease in the Seebeck coefficient, keeping the *S* values of heavily doped phases at relatively high levels. Interestingly, density functional theory (DFT) calculations, using the Korringa−Kohn−Rostoker method with the coherent potential approximation (KKR-CPA) method to account for such a complex chemical disorder, reveal aspects related to the role and unique contribution of Ti in the formation of the electronic structure of the (Nb,Ta)­FeSb system in the vicinity of the Fermi level (*E*_F_). Ti substitution seems to contribute to the sharpness enhancement of the total electronic density of states (DOS) close to *E*_F_, since Ti presents a higher and sharper DOS than those of Nb and Ta. According to the Mott relation, this probably affects positively the thermopower of heavily doped phases. Theoretical estimations of the Seebeck coefficient based on the electronic DOS, calculated by the KKR-CPA method, tend to support this evidence. In addition, Ti substitution in combination with Nb/Ta alloying and nanostructuring induces an effective phonon scattering, strongly reducing the lattice thermal conductivity. Theoretical calculations of lattice thermal conductivity are in very good agreement with the experimental measurements, revealing the strong effect of Ti on the enhancement of mass fluctuation scattering and the reduction of the phonon mean free path. As a result, a powerful enhancement of *ZT* is achieved for Nb_0.45_Ti_0.15_Ta_0.4_FeSb, reaching a maximum value of 1 at 600 °C, and highlighting the multiple roles of Ti in the improvement of thermoelectric (TE) performance of *M*_V_FeSb-based HH phases.

## Graphical abstract

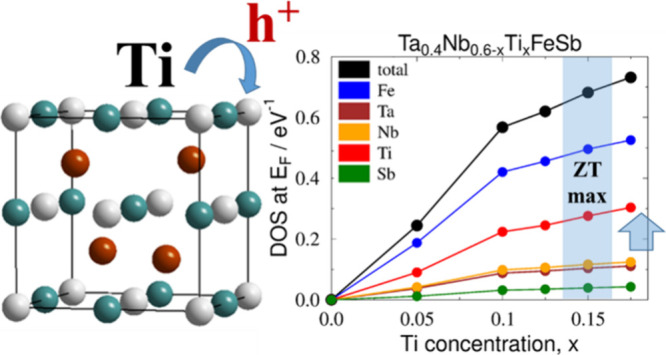

## Introduction

Considering that almost 66% of global energy consumption is emitted to the environment as waste heat, TE power generation can provide a powerful green energy solution for energy harvesting and waste heat recovery, effectively contributing to power production.^1,2^ Thermoelectric generators (TEGs) are able to produce clean electrical power if a temperature gradient is applied at their ends. The operation of TEGs is closely related to the underlying TE properties of materials, which are classified according to the temperature range in which they exhibit their optimum efficiency.^3^ The TE performance of materials is determined by the dimensionless figure of merit, *ZT* = *S* ^2^σ*T*/κ, which is dependent on the electrical conductivity (σ), Seebeck coefficient (*S*), and thermal conductivity (κ), with the optimum reached for a specific range of temperature (*T*). A maximum TE efficiency requires the optimization of electrical transport properties in order to achieve a high power factor PF = *S* ^2^σ as well as a low thermal conductivity.

Commercially available TEGs are made of Bi_2_Te_3_ materials, which efficiently operate in the temperature range of 300−500 K,^3^ while PbTe,^4^ and GeTe^5^ compounds maximize their performance at higher temperatures. However, tellurides are not appropriate for large-scale applications since tellurium is a high-cost and scarce material.^6^ Other materials such as silicides,^7,8^ oxides,^9^ and skutterudides^10^ have shown promising TE properties for intermediate temperature applications. Intensive investigations have been performed in recent years to discover high-performance, environmentally friendly, and low-cost materials for thermoelectricity generation.^11,12^ New design strategies such as band structure engineering,^13,14^ the panoscopic approach,^15,16^ and all-scale hierarchical phonon scattering architectures^4^ have led to remarkable improvements of *ZT*, opening the road for future commercialization.^17^

Recently, the group of HH phases has attracted considerable research interest, since these materials meet a wide range of criteria that make them leading TE candidates for intermediate and high-temperature applications, close to the temperature range of most industrial processes.^18–20^ They exhibit promising electrical transport properties but also exceptional mechanical properties, nontoxicity, and good chemical stability at high temperatures.^18,21–23^ HH semiconducting phases are intermetallic compounds with a valence electron count of 18 and the general formula *MM'X*, which are crystallized in the cubic MgAgAs-type structure with the space group *F*4̅3*m*. Until recently, most investigations were focused on the n-type *M*NiSn^24–29^ and p-type *M*CoSb^30–33^ phases (*M* = Ti, Zr, Hf). A wide range of chemical substitution studies have been attempted at the three atomic positions to optimize their TE properties. Complex structures such as Ti_0.5_Zr_0.5_NiSn_0.98_Sb_0.02_, Zr_0.75_Hf_0.25_NiSn_0.99_Sb_0.01_, and Ti_0.57_Zr_0.4_Al_0.02_Ta_0.01_NiSn_0.98_Sb_0.02_ demonstrate the best n-type TE efficiencies, reaching *ZT* values of 1−1.4,^34–38^ while p-type phases such as Ti_0.8_Hf_0.2_Fe_0.5_Co_0.15_Ni_0.35_Sb and Hf_0.6_Ti_0.4_CoSb_0.83_Sn_0.17_ exhibit a maximum *ZT* with a range of 0.8−1.1.^39,40^ Moreover, great advances have been made on HH compounds in the last ten years, and new highly promising p-type phases based on *M*_V_FeSb (*M*_V_ = V, Nb, Ta) and ZrCoBi have been discovered.^41^

The Hf-free *M*_V_FeSb compounds have recently been considered as the most attractive p-type HH phases due to their outstanding electronic properties, superior TE efficiencies, and competitive cost. NbFeSb-based phases are heavy-band semiconductors which allow large amounts of dopants for the optimization of electrical transport properties.^42^ Although the optimum carrier concentration for these compounds is almost 1 order of magnitude higher than those of other state-of-the-art TE materials (with *n* ∼ 10^19^−10^20^ cm^−3^), they still exhibit relatively large values of Seebeck coefficient due to the high band degeneracy.^43^ First experimental and computational studies revealed the great effect of chemical substitutions on the TE properties of *M*_V_FeSb (*M*_V_ = V, Nb) phases.^44,45^ Specifically, by implementing appropriate hole doping, these compounds can reach very high power factors.^42,46–49^ Band engineering is a powerful strategy that can significantly enhance the TE performance of materials, closely related to the quality factor *B* ∝ *N*_v_/*m*_b_*κ_lat_, where *N*_v_ is the number of degenerate valleys and *m*_b_* is the band effective mass.^50,51^ Therefore, a large number of degenerate valleys in combination with a low *m*_b_* and reduced lattice thermal conductivity (κ_lat_) can lead to great enhancements in TE figure of merit. A band degeneracy of 8 was shown for the Ti-doped FeV_0.6_Nb_0.4_Sb solid solution, reaching a *ZT* of 0.8 at 900 K.^43^ On the other hand, the V-free Nb_1−*x*_Ti_*x*_FeSb system exhibits a better carrier mobility than that of the V/Nb solid solution, due to the reduction of band effective mass, resulting in a *ZT* of 1.1 at 1100 K.^51^ Implementing Hf doping in NbFeSb, a significant maximum *ZT* of ∼1.50 was achieved at 1200 K.^42^ However, Ta has been proven to be the most powerful player in the TE performance competition of p-type *M*_V_FeSb phases. Band structure calculations revealed an effective convergence of valence bands for TaFeSb, also resulting in a valley degeneracy of 8. Implementing Ti doping led to quite large PF values, and a record high *ZT* of 1.52 was achieved for the Ta_0.74_V_0.1_Ti_0.16_FeSb solid solution at 973 K.^52^ In addition, Yu et al. showed that Nb/Ta alloying causes an effective phonon scattering, resulting in a strong reduction of lattice thermal conductivity and a maximum *ZT* of 1.6 at 1200 K.^53^ Recently, the development of Ta_0.42_Nb_0.3_V_0.15_Ti_0.13_FeSb-(InSb)_0.015_ composite led to an appreciable enhancement of PF, reaching a maximum *ZT* of 1.43 at 973 K.^54^ Moreover, Zhu et al. showed that the Sb-pressure-controlled annealing process of Nb_0.55_Ti_0.05_Ta_0.4_FeSb modulates the microstructure of the material, which greatly affects the hole mobility and electrical conductivity, resulting in a remarkable enhancement of PF with values of 78 μW cm^−1^ K^−2^ close to room temperature.^46^ Ti has been proven a powerful dopant for the NbFeSb-based systems, causing an effective increase in hole concentration and electrical conductivity. However, very few studies have investigated the effect of Ti doping in the formation of electronic DOS in the (Nb,Ta)­FeSb system.^55,45^

In the current study, HH phases of the general formula Nb_0.6−*x*_Ti_*x*_Ta_0.4_FeSb (0 ≤ *x* ≤ 0.175) were synthesized by mechanical alloying (MA) using a straightforward and single-step ball milling process. The effect of Ti doping on the TE properties of prepared materials is investigated, combining experimental and computational methods. DFT calculations reveal the important role of Ti in the formation of the electronic structure of the (Nb,Ta)­FeSb system. Ti substitution seems to further enhance the sharpness of the DOS close to E_F_. Interestingly, the strong increase in electrical conductivity which comes from the effective hole doping of Ti substitution is not accompanied by a corresponding reduction in the Seebeck coefficient. Experimental results along with theoretical estimations of thermopower support the suggestion that Ti may contribute to the sharpness of DOS at E_F_, compensating for the increase in hole concentration and thus maintaining the thermopower at relatively high levels for the Ti-rich phases. In addition, the combination of Ti point defects, Nb/Ta alloying, and nanostructuring effect results in an ultralow lattice thermal conductivity which, in combination with the obtained high PF, greatly enhances the TE figure of merit of materials.

## Materials and Methods

Nb_0.6−*x*_Ti_*x*_Ta_0.4_FeSb (0 ≤ *x* ≤ 0.175) were synthesized by MA. Appropriate stoichiometric amounts of reagent elements with purity greater than 99.8% from Alfa Aesar were mixed and sealed in a stainless steel vial under an argon atmosphere with a ball to powder ratio of 10:1. MA was carried out at 450 rpm for 24 h in a planetary mill (Pulverisette 6, Fritsch).

Powder X-ray diffraction (XRD) measurements were carried out using a Rigaku SmartLab diffractometer which operates with a Cu−Kα source at 9 KW (45 kV, 200 mA). A scan time of 0.6 s per step and a scan step of 0.02° over the angular range 10 ≤ 2θ/° ≤ 90 were set. The Rietveld method was performed using the General Structure Analysis System (GSAS) software package.

Samples for electron microscopy characterization (transmission electron microscopy (TEM), high-resolution transmission electron microscopy (HRTEM), high-angle annular dark-field scanning-transmission electron microscopy (HAADF-STEM), and electron energy loss spectroscopy (EELS)) were prepared by gently grinding the material powders in high-purity ethanol using an agate pestle and mortar. A drop of the solution was subsequently deposited onto a lacey carbon film supported on a Cu grid and allowed to evaporate under ambient conditions. Electron microscopy experiments were carried out in a Thermo Fischer Scientific Talos F200i HRSTEM operating at 200 kV, equipped with an X-CFEG electron source and with a STEM point resolution of 0.14 nm. HAADF-STEM images were acquired on a Fischione Model 3000 HAADF detector with a camera length such that the inner cutoff angle of the detector was 50 mrad. Elemental analysis was performed by means of energy-dispersive X-ray spectroscopy (EDS), using a Bruker XFlash 6−100 spectrometer with an energy resolution of 127.9 eV (Mn K_α_ line).

DFT electronic structure calculations were performed using the Green function self-consistent Korringa−Kohn−Rostoker method with coherent potential approximation (KKR-CPA).^56,57^ The CPA model appears to be particularly well-adapted to study highly disordered materials, since it allows to account for ab initio computations of random atom distributions on selected crystallographic sites. The translational symmetry lost in alloys is recovered by applying the effective medium (CPA) defined by the Green function *G*_cpa_ = Σ_*i*_ *x_i_G_i_* as obtained from the averaging of Green’s functions ascribed to particular atoms *G_i_* over their concentrations *x_i_* (Σ_*i*_ *x_i_* = 1). In the investigated Nb_0.6−*x*_Ti_*x*_Ta_0.4_FeSb system, the “disordered site” is replaced by *G*_cpa_ = (0.6 − *x*)*G*_Nb_ + *xG*_Ti_ + 0.4*G*_Ta_, whereas two other “ordered sites”, Fe and Sb, are defined by the Green functions G_Fe_ and G_Sb_, respectively. The CPA condition is solved self-consistently. It is worth noting that within the CPA model (unlike the supercell approach), the symmetry of the unit cell is maintained in any range of alloy composition, for which ground state properties can be computed (charges, densities of states, total energy, etc.). The self-consistent crystal potential was constructed within the local density approximation (LDA), using the Perdew−Wang formula for the exchange-correlation part. The KKR-CPA computations in the disordered Nb_0.6−*x*_Ti_*x*_Ta_0.4_FeSb used the muffin-tin form of crystal potential with truncations on each atom up to the angular momentum cutoff *l*_max_ = 3. For well-converged atomic charges (below 10^−3^ *e*) and crystal potentials (below 10 meV), the total, site-decomposed and *l*-decomposed DOS were computed using the integration tetrahedron method in reciprocal space and 560 k-space points in the irreducible part of the Brillouin zone. The Fermi level was accurately determined from the Lloyd formula.^58^ All calculations were performed for the nominal compositions, assuming experimental values of lattice parameter, but they were also determined from the KKR-CPA by minimizing total energy. The theoretical value of the Seebeck coefficient *S* was evaluated assuming dominance of the electron-diffusion contribution and negligible phonon-drag effects. Within this approximation, the electrical conductivity can be expressed in terms of the electronic DOS, and the thermoelectric coefficient, *S*/*T*, can be estimated using the simplified Mott formula (eq 1).^59^

$$\frac{S}{T}=\frac{\pi^{2}}{3}\frac{k_{B}^{2}}{e}\frac{1}{N_{d}(E_{F})}\frac{dN_{d}(E)}{dE}{|}_{E_{F}}$$ 1

*N*_d_(*E*) is the energy-dependent total DOS function of the system, *k*_B_ is the Boltzmann constant, and *e* is the electron charge. The linear part of thermopower is numerically derived at *E* = *E*_F_ and then multiplied by any temperature.

The lattice thermal conductivities were computed from *ab initio* calculations using Phono3py.^60,61^ All DFT calculations were performed using the Vienna ab initio simulation package (VASP),^62,63^ employing the PW92 ^64^ LDA and a 450 eV planewave energy cutoff. Phonon properties were calculated using the finite displacement method in a 2 × 2 × 2 supercell containing 96 atoms. The corresponding Brillouin zone was sampled using a 2 × 2 × 2 mesh. Harmonic phonon properties were calculated using Phonopy.^61^ The lattice thermal conductivity was evaluated by solving the phonon Boltzmann transport equation under a relaxation-time approximation. To compute the lattice thermal conductivity, the Brillouin zone was sampled using a 30 × 30 × 30 *q*-point mesh.

In Phono3py calculations, the typical workflow is to compute the harmonic and anharmonic force constants from the above-mentioned VASP calculations. To be fully consistent with the computation of the electronic properties reported in this paper, one should also use the CPA approximation to compute the force constants of the Nb_0.6−*x*_Ti_*x*_Ta_0.4_FeSb alloys. This approach is, however, not available in VASP. Instead, we will use the simpler virtual crystal approximation (VCA),^65,66^ where the average of the Hamiltonian is considered rather than the average of the Green’s function. Because typical phonons have a much larger wavelength than electrons, the VCA is usually much better for phonon properties than for electrons. When the phonons obtained from those average force constants are propagating in the alloys, they may collide with other phonons. Therefore, the relaxation time used to solve the Boltzmann equation must contain phonon−phonon interactions. However, when those average phonons approach the atomic site experiencing disorder (with Wyckoff position 4*a*), they are also scattered by the mass fluctuation originating from the disorder. We have included mass fluctuation scattering in our calculations, following the Tamura model.^67^

The densification of developed powders into pellets was carried out uniaxially through hot-press sintering under argon atmosphere at 810 °C with a pressure of 80 MPa for 1h in a HP20, Thermal Technologies system. The experimental density of pellets (ρ) was calculated by the geometrical method. Using a ZEM-3 ULVAC-RIKO electrical conductivity (σ) and Seebeck coefficient (*S*) measurements were carried out under a helium atmosphere in the temperature range 300 K ≤ *T* ≤ 773 K. The thermal diffusivity (*D*) and specific heat capacity (*C*_p_) of the samples were measured by a Netzsch LFA 457 laser setup. Data were collected in 50 K increments on pellets coated with graphite. A pyroceram reference sample was used for the *C*_p_ measurements. The thermal conductivity was determined by using the formula κ = *D*ρ*C*_p_. Estimated uncertainties for the measurements of electrical and thermal transport properties are ±5 and ±7%, respectively.

## Results and Discussion

HH compounds with the general formula Nb_0.6−*x*_Ti_*x*_Ta_0.4_FeSb (0 ≤ *x* ≤ 0.175) were synthesized by MA and hot-press sintering. Powder XRD measurements were performed to test the purity of prepared phases and study their crystal structure. As shown in Figure 1, all products of the series Nb_0.6__−__*x*_Ti_*x*_Ta_0.4_FeSb (0 ≤ *x* ≤ 0.175) exhibit high purity and possess the HH phase as a single one without evidence for secondary phases or unreacted reagent elements. This is in agreement with previous studies which have also shown high purity in Ti-doped NbFeSb phases, even with a higher Ti content.^48,51^ Rietveld refinements were executed for Nb_0.6__−*x*_Ti_*x*_Ta_0.4_FeSb (0 ≤ *x* ≤ 0.175) phases to investigate their structural properties. Figure 2 presents the refinement profile for *x* = 0.15, while the remaining profiles are presented in Supporting Information (Figures S1−S5). A quite good fitting of experimental data with the calculated model can be observed, validating the cubic structure of HH phases with space group *F*4̅3*m*. It must be mentioned that Rietveld refinements did not show evidence for any antisite defects or vacancies in all crystallographic sites. Ti atoms occupy, along with Nb and Ta ones, the Wyckoff site 4*a* (0 0 0), while Fe and Sb atoms occupy the 4*c* (141414) and 4*b* (121212) sites, respectively. As shown in Table 1, Ti substitution does not seem to cause noticeable changes in the unit cell across the series Nb_0.6__−*x*_Ti_*x*_Ta_0.4_FeSb (0 ≤ *x* ≤ 0.175). The values of the lattice constant derived by powder XRD Rietveld refinements are in good agreement with the theoretical results obtained from the KKR-CPA calculations (Figure S6), showing that the lattice parameter remains more or less constant with Ti increase since the empirical atomic radius of Ti (*r*_Ti_ = 140 pm) is quite close to that of Nb (*r*_Nb_ = 145 pm).

**Figure 1:**
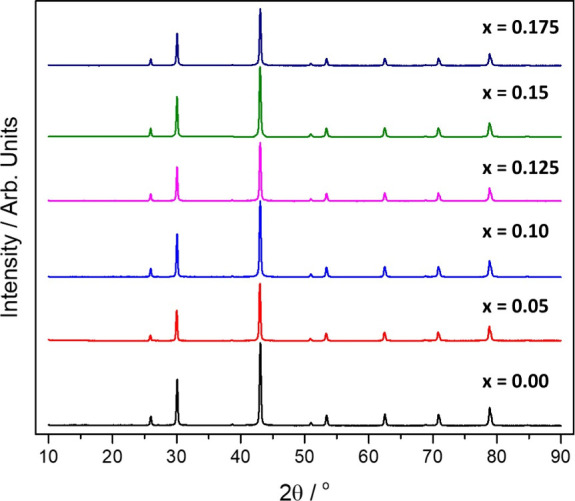
Powder XRD data in arbitrary units for the Nb_0.6__−*x*_Ti_*x*_Ta_0.4_FeSb (0 ≤ *x* ≤ 0.175) series in the angle range 10 ≤ 2θ/° ≤ 90.

**Figure 2:**
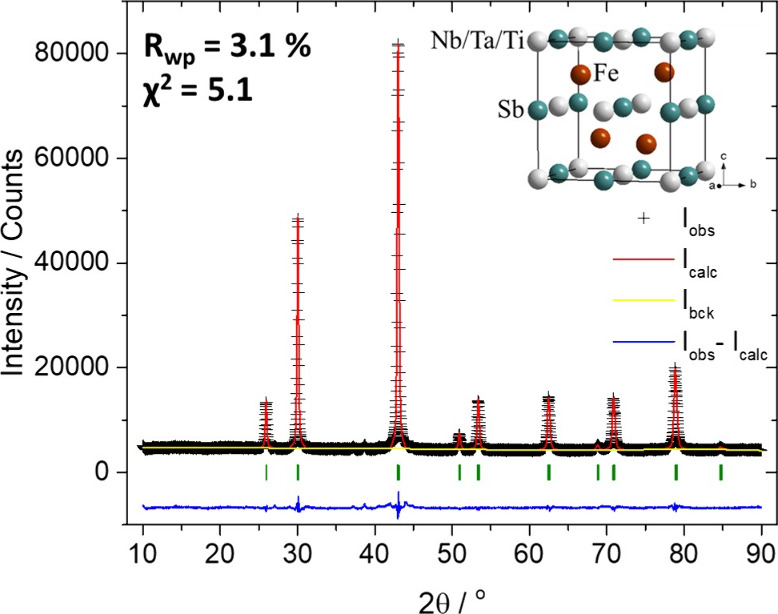
Powder XRD Rietveld refinement profile for the Nb_0.45_Ti_0.15_Ta_0.4_FeSb: final observed (black crosses), calculated (red solid line), calculated background (yellow line), and difference (blue line). Reflection positions for the HH phase are marked with olive color. Inset: the cubic structure of HH phases with space group F4®3m .

**Table 1:** Lattice Parameters of Nb_0.6−*x*_Ti_*x*_Ta_0.4_FeSb (0 ≤ *x* ≤ 0.175) Extracted from Powder XRD Rietveld Analysis and DFT Calculations Using the KKR-CPA Method

*x*	KKR-CPA *a*/Å	XRD *a*/Å	XRD V/Å^3^
0	5.936(5)	5.94353(2)	209.958(3)
0.05	5.943(9)	5.94287(3)	209.888(3)
0.10	5.943(3)	5.94388(4)	209.996(4)
0.125	5.943(2)	5.94276(2)	209.877(3)
0.15	5.939(5)	5.94339(3)	209.944(4)
0.175	5.944(3)	5.94285(2)	209.886(3)

A representative sample, in the middle of the *x*-range values (*x* = 0.1) of the Nb_0.6−*x*_Ti_*x*_Ta_0.4_FeSb series, has been selected for electron microscopy investigations. The typical morphology and particle size range of the Nb_0.5_Ti_0.1_Ta_0.4_FeSb sample are illustrated in the conventional TEM image of Figure 3a. The powder particles appear evenly dispersed and with no specific shape. They possess an average size of 172 nm, ranging from 111 to 267 nm. The average grain size of nanopowders obtained in this study is lower, compared to the ∼250 nm of Ti-doped TaFeSb phases reported by Zhu et al.,^52^ since different ball milling conditions were applied. The corresponding inset Selected Area Diffraction (SAD) pattern in Figure 3a, viewed along the [011] zone axis, confirms the single crystalline nature of the HH particles, in line with the XRD results. Detailed measurements of the *d* lattice spacings from such SAD patterns predominantly assigned them to the mixed Nb_0.6_Ta_0.4_FeSb HH phase; this additionally confirms that the Nb_0.5_Ti_0.1_Ta_0.4_FeSb sample is a single phase. The average lattice constant is *a*_SAD_ = 0.5996 nm, ranging from 0.594 to 0.6033 nm. For comparison reasons, the experimental lattice constant from SAD complies well with both values derived by the XRD Rietveld analysis (*a*_XRD_ = 0.5944 nm) and DFT calculations (*a*_DFT_ = 0.5943 nm).

**Figure 3:**
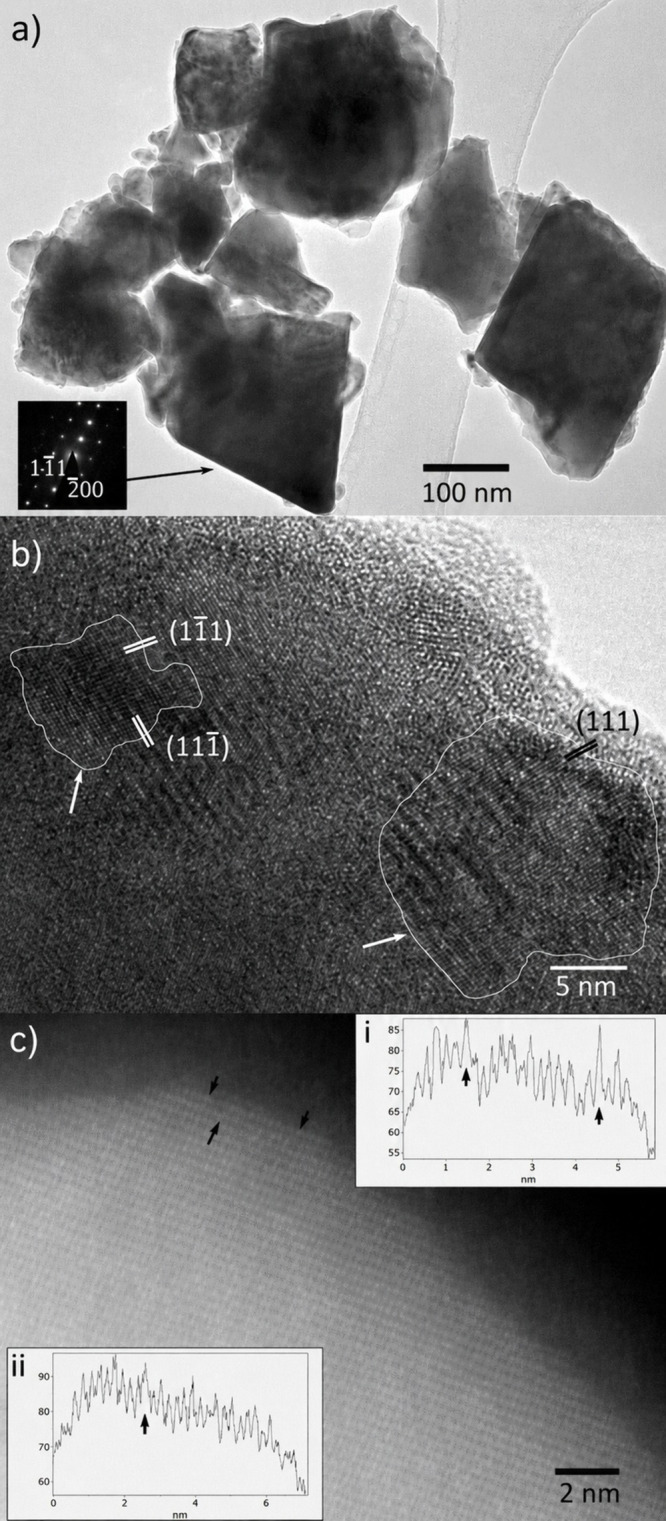
Electron microscopy results from the 10 at % Ti-doped sample: (a) conventional TEM image and [011] SAD pattern inset, coming from the arrowed HH particle; (b) HRTEM image of small nanoparticles; (c) HAADF image from the periphery of a typical HH particle. The two insets, i and ii, show the intensity distributions of the first and second atomic rows, respectively, where certain variations are revealed, implying different *Z* elements in the respective columns pointed with black arrows.

Along with the large particles, the sample contains a significant amount of nanograin formation, agglomerated with the larger ones of Figure 3a. The nanograin morphology is best illustrated in the HRTEM image of Figure 3b. In line with the large particles, they also exhibit high crystallinity, as shown by the {111} lattice fringes revealed in the HRTEM image. Measurements of the nanoparticle size distributions from HRTEM images resulted in 10−25 nm. Detailed measurements of the lattice fringe spacings in similar HRTEM images have been performed in order to derive the “nanometer” scale lattice constant (*a*_HRTEM_), which was *a*_HRTEM_ = 0.5954 nm, with a range of 0.5847−0.6025 nm. Although the range of values derived from HRTEM is greater than the SAD patterns, the average value is remarkably close to both the experimental one measured by XRD and theoretically calculated by DFT. Furthermore, the coexistence of numerous large particles, on the one hand, and, especially, nanoparticles, on the other, in dense configurations merely results in a large number of grain boundaries that effectively act as scattering centers for phonons, leading thus to a reduction in lattice thermal conductivity, as TE property measurements prove, too. We will see in the following, however, that the effectiveness of one kind of particle or the other depends on the Ti concentration.

Ti incorporation in the HH lattice has been also manifested by HRSTEM imaging experiments. Figure 3c shows a high angle annular dark field (HAADF) image of a typical HH particle. The inset intensity distributions, i and ii, along the atomic columns at the near surface region of the particle reveal characteristic intensity variations, which can be attributed to the existence of lower (Ti) and higher (Ta) Z kinds of atoms in the HH lattice, in random distribution. This has been also confirmed by EDS results, where the average Ti, Nb, and Ta contents exhibited certain variations between distinct HH particles, both large ones and nanoparticles alike. It has to be noted that elemental fluctuations of the other site elements (Fe and Sb) are not profound, in line with the Rietveld analysis findings. This further confirms Nb/Ta alloying, enhanced by Ti incorporation in the lattice, having a positive effect on lattice thermal conductivity reduction.

The KKR-CPA calculations of Nb_0.6−*x*_Ti_*x*_Ta_0.4_FeSb HH systems allowed us to determine DOS for the experimental compositions *x* = 0, 0.05, 0.10, and 0.15. Electronic DOS diagrams as a function of energy for Nb_0.6−*x*_Ti_*x*_Ta_0.4_FeSb phases are illustrated in Figure 4. As can be observed, the pristine phase is a narrow-band gap semiconductor with the Fermi level at the valence band edge. As expected, *E*_F_ is gradually shifted deeper into the valence states with the increase of the Ti content, leaving more unoccupied orbitals. The analysis of carrier concentration from DOS also confirms that titanium behaves as the effective one-hole donor, since Ti (*Z* = 22) is one electron-deficient element compared to the substituted Nb (*Z* = 41) and Ta (*Z* = 73), which are isovalent. This behavior will be shown later in the electrical transport property measurements. The electronic structure of (Nb−Ta)­FeSb HH is built of d-states of transition-metal atoms, being strongly hybridized with p-states of Sb, which in consequence leads to the formation of the energy gap above the ninth valence band (VEC = 18). The strongest contribution to the total DOS of the valence band comes from d-states of Fe, while elements from group V (Nb, Ta) present moderate contributions with strikingly similar DOS curves. Interestingly, in the vicinity of the Fermi energy, Ti exhibits a higher and sharper site-decomposed DOS than those of Nb and Ta. The previous study of Zhu et al. revealed a high band degeneracy in the electronic structure of the TaFeSb system, which is highly advantageous to achieve high PF values.^52^ The high band degeneracy and consequently the large slope of total DOS close to *E*_F_ may be the reason for the high Seebeck coefficient values observed at high carrier concentrations.^43^ In Figure S7, electronic band structure calculations for Nb_0.6_Ta_0.4_FeSb and Nb_0.45_Ti_0.15_Ta_0.4_FeSb phases show that the valence band maxima (VBM) are located at the L point of the Brillouin zone and exhibit a 2-fold orbital degeneracy, confirming previous studies.^48,52,55,68^ This band convergence leads to a high valley degeneracy *N*_v_ of 8,^52^ enhancing the DOS near the Fermi level and effectively contributing to the improvement of the TE performance.

**Figure 4:**
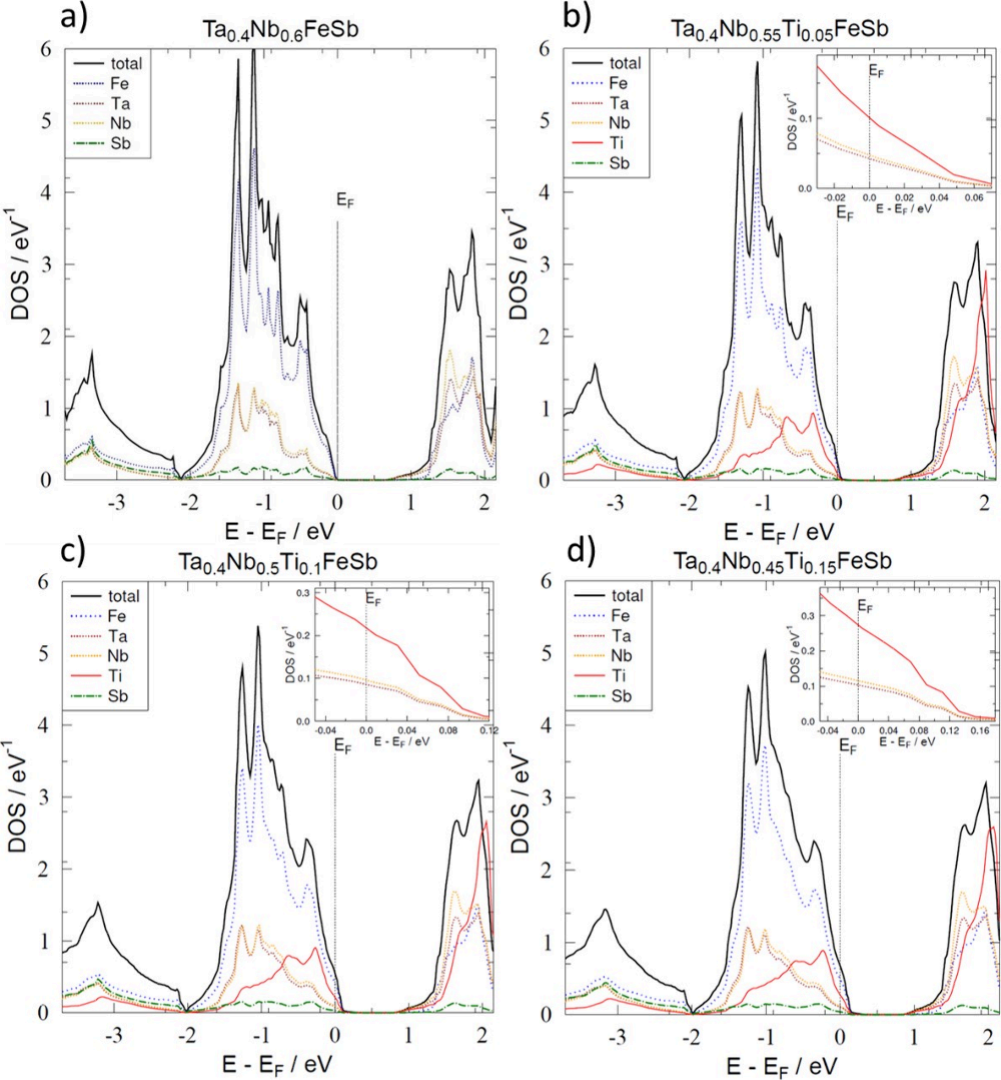
Evolution of total and site-decomposed electronic DOS in Nb_0.6−*x*_Ti_*x*_Ta_0.4_FeSb HH phases for *x* = 0 (a), 0.05 (b), 0.10 (c), and 0.15 (d), as calculated by the KKR-CPA method. The DOS contributions from atoms (Nb, Ta, Ti) on the “disordered site” zoomed near *E*_F_ are also presented in insets. The Fermi energy is shifted to zero.

Until now, it was believed that Ti substitution has no effect on the formation of band structure.^51^ The domination of the Fe character in the electronic DOS overshadowed the contributions of the remaining atoms. Here, the unique role of Ti in the modification of the electronic structure of (Ta,Nb)­FeSb system is revealed. This is clearly observed in Figure S8 which shows the total and site-decomposed DOS at E_F_ in Nb_0.6__−*x*_Ti_*x*_Ta_0.4_FeSb as a function of Ti content. The contribution of Ti into the total DOS makes it possible to enhance its slope sharpness near *E*_F_, especially for the Ti-rich phases with *x* ≥ 0.1. According to the Mott relation, this could enhance the DOS derivative at the *E*_F_, maintaining the thermopower at high levels even for the Ti rich-doped HH phases with high carrier concentrations. The KKR-CPA calculations in Figure S7 further indicate that Ti substitution modifies the dispersion of the valence bands near the VBM. In the present analysis, the real part of the CPA-derived band structure is used to evaluate the band curvature near the VBM. Within this framework, the increasing sharpness of the DOS near the Fermi level with Ti content is consistent with a reduction of the band curvature (d^2^ *E*/d*k* ^2^), i.e., band flattening. Using a parabolic approximation around the VBM, we estimated the band effective mass (*m*_b_*) (Figure S7, insets). The results indicate that *m*_b_* increases with Ti concentration, in agreement with the KKR-CPA-derived band dispersion, confirming a progressive flattening of the valence bands near the VBM. As a consequence, the enhanced band effective mass contributes to an increased DOS effective mass via the formula *m*_DOS_* = *N*_v_^2/3^ *m*_b_*,^54^ providing a consistent explanation for the relatively high Seebeck coefficient, as observed later in Ti-rich compositions, despite the increased carrier concentration.

After the fabrication of high-density pellets by using hot-press sintering, electrical and thermal transport property measurements followed. It must be noted here that all samples exhibit experimental densities above 97% of the theoretical values, as calculated by Rietveld analysis (Table S1). Electrical conductivity and Seebeck coefficient data as a function of temperature are presented in Figure 5. Ti substitution induces an effective hole doping in the system, significantly increasing the electrical conductivity of materials. It is notable that for the heavily doped phases, *x* ≥ 0.10, the materials present a degenerate semiconducting behavior with high electrical conductivity values close to room temperature. As shown in previous studies, the characteristic reduction in σ as a function of temperature indicates that acoustic phonon scattering dominates in charge transport,^42,43,51,69^ while the atomic disorder coming from Ti substitution or Nb/Ta alloying scattering should also affect to an extent the carrier transport. In addition, it must be mentioned that the increased *m*_b_* observed previously in the Ti-rich phase (Figure S7) may be responsible for a reduction in hole mobility as Ti content increases. This seems to explain the fact that the increase rate of electrical conductivity shows a gradual decline for the Ti-rich phases with *x* ≥ 0.1. It is characteristic that the phases with the highest Ti content (*x* = 0.15 and 0.175) present σ values quite close to each other. On the other hand, the pristine material exhibits a poor electrical conductivity, highlighting the strong impact of Ti on the increase of hole concentration in these heavy-band p-type semiconductors. Similar behavior of electrical conductivity with Ti doping has been observed for TaFeSb and NbFeSb systems in previous studies,^47,52^ where Hall effect measurements confirmed the enhancement of carrier concentration with the increase of Ti content.

**Figure 5:**
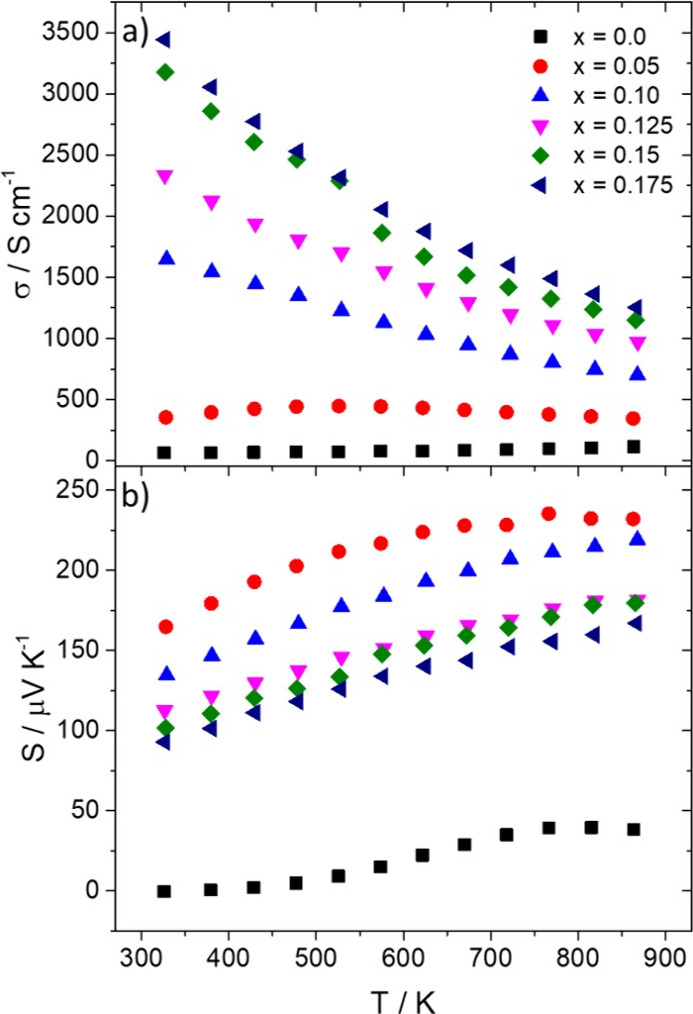
(a) Electrical conductivity and (b) Seebeck coefficient as a function of temperature for Nb_0.6__−*x*_Ti_*x*_Ta_0.4_FeSb (0 ≤ *x* ≤ 0.175) phases.

Seebeck coefficient measurements of Ti-doped phases show a gradual reduction with the increase of Ti content, which is indicative of the increase in hole concentration. For the pristine phase, Nb_0.6_Ta_0.4_FeSb, the *S* values are quite low and exhibit a similar trend to that of the TaFeSb system.^52^ However, due to the increased Nb content, Nb_0.6_Ta_0.4_FeSb denotes bipolar conduction, exhibiting a marginal negative value close to room temperature, as NbFeSb.^69^ It is noteworthy that the Ti-rich phases present *S* values quite close to each other for *x* ≥ 0.125. In order to extract valuable conclusions about the effect of Ti doping on the electrical transport properties, the Seebeck coefficient and electrical conductivity measurements at a constant temperature are presented in Figure 6 as a function of Ti content. The experimental results of the Seebeck coefficient (*S*_exp_) are also compared with theoretical values (*S*_theor_) calculated by using the simplified Mott formula, where the linear part is estimated from the DOS derivative at the *E*_F_.

**Figure 6:**
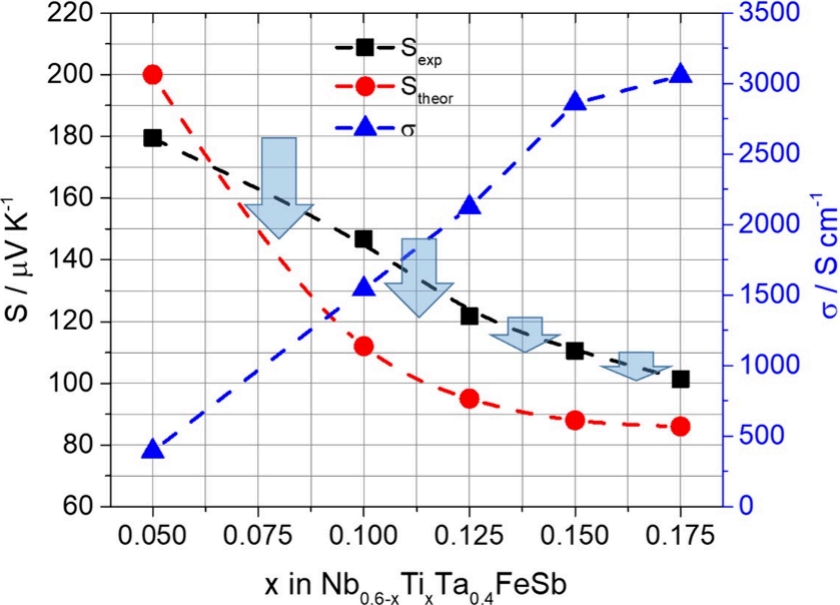
Measured thermopower (*S*_exp_) of Nb_0.6__−*x*_Ti_*x*_Ta_0.4_FeSb (0.05 ≤ *x* ≤ 0.175) at 400 K as a function of Ti content *x*, compared to theoretical predictions from the Mott formula (*S*_theor_) relying on KKR-CPA DOS. The right axis depicts electrical conductivity measurements at 400 K as a function of Ti content.

For most of the Ti-doped phases, the experimental measurements are in reasonable agreement with the theoretical estimations of the Seebeck coefficient. Interestingly, both *S*_exp_ and *S*_theor_ results show that the decrease rate in the Seebeck coefficient (light blue arrow) is reduced gradually with the increase of Ti content. On the other hand, electrical conductivity shows almost a linear increase with the Ti content. It is notable that the Ti-doped phase with *x* = 0.15 reaches a *S* value close to that of *x* = 0.125, while in parallel, it exhibits an appreciable increase in electrical conductivity. The reduction of slope in electrical conductivity for *x* = 0.175 may also be indicative of a slight decline of hole mobility due to the increase of *m*_b_*. However, the system seems to preserve an adequate carrier mobility up to *x* = 0.15 due to the weak phonon scattering and the low scattering potential coming from the atomic disorder and alloying as derived by Fu et al.^43^ The aforementioned results in electrical transport properties together with the previous evidence in DOS outputs from KKR-CPA calculations support our suggestion that *E*_F_ may be shifted to a sharp region in the electronic DOS. In order to avoid any confusion, it must be mentioned here that the predominant contribution in the formation of total DOS comes from Fe (Figure S8). However, the substitution of Nb by Ti atoms may further enhance the sharpness of the total DOS curve due to the stronger contribution in the region close to *E*_F_. As a result, the shift of *E*_F_ in a sharper region of the valence band may compensate for the increase in hole concentration due to Ti doping. This suggestion seems to explain the moderate reduction in the Seebeck coefficient observed for the Ti-rich phases, while the electrical conductivity continues to increase sufficiently up to *x* = 0.15.

Figure 7 presents the PF of Nb_0.6__−*x*_Ti_*x*_Ta_0.4_FeSb series as a function of temperature. As expected, the heavily doped phases with *x* ≥ 0.10 exhibit a significant improvement of PF, compared with the pristine and low Ti doping (5 at %) samples. The optimization of electrical transport properties is achieved for Nb_0.45_Ti_0.15_Ta_0.4_FeSb phase which demonstrates the highest PF across the whole temperature range and a maximum value close to 41 μW cm^−1^ K^−2^ at 525 K. This notable TE performance is attributed to the significant increase in electrical conductivity, but also quite important is the moderate reduction in Seebeck coefficient.

**Figure 7:**
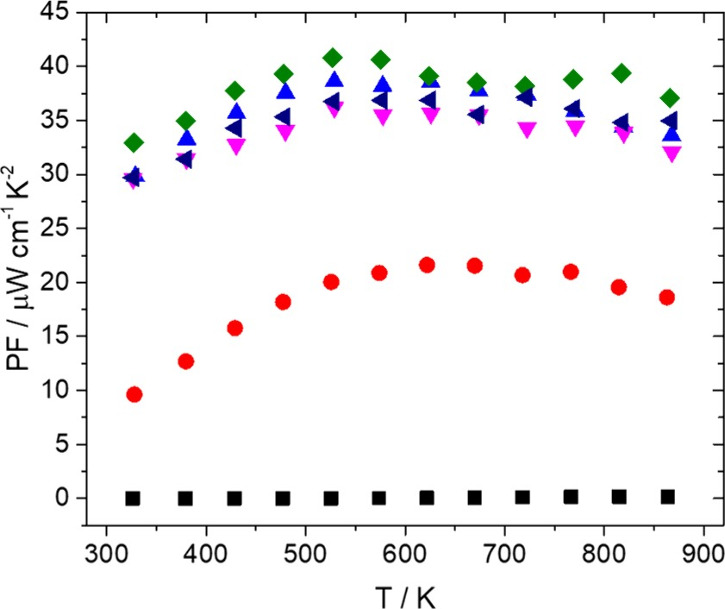
PF as a function of temperature for the Nb_0.6−__*x*_Ti_*x*_Ta_0.4_FeSb (0 ≤ *x* ≤ 0.175) series.

Figure 8 shows total thermal conductivity (κ_tot_) measurements as well as the lattice (κ_lat_) contribution as a function of temperature. It must be noticed here that the electronic contribution to thermal conductivity was estimated by using the Wiedemann−Franz law. The Lorenz number was calculated by using Fermi−Dirac statistics and the Seebeck coefficient data, considering predominant scattering from acoustic phonons.^70,71^ Subtracting the electronic contribution from the total thermal conductivity (κ_tot_ − κ_el_), κ_lat_ was determined. As shown in Figure 8a, there is a clear difference in total thermal conductivity of pristine material with those of Ti-doped phases, due to the very low electronic contribution of first one. Comparing the values of Ti-doped phases, it is obvious that the total thermal conductivity remains almost at the same levels across the temperature range and across the series without great changes. On the other hand, Figure 8b shows a gradual reduction in lattice thermal conductivity with the increase in Ti content for *x* ≥ 0.1. Therefore, it is concluded that the reduction in κ_lat_ for the Ti doped-phases seems to counterbalance the increase in electronic contribution, keeping the total thermal conductivity at low levels, without increasing with the increase of charge carriers.

**Figure 8:**
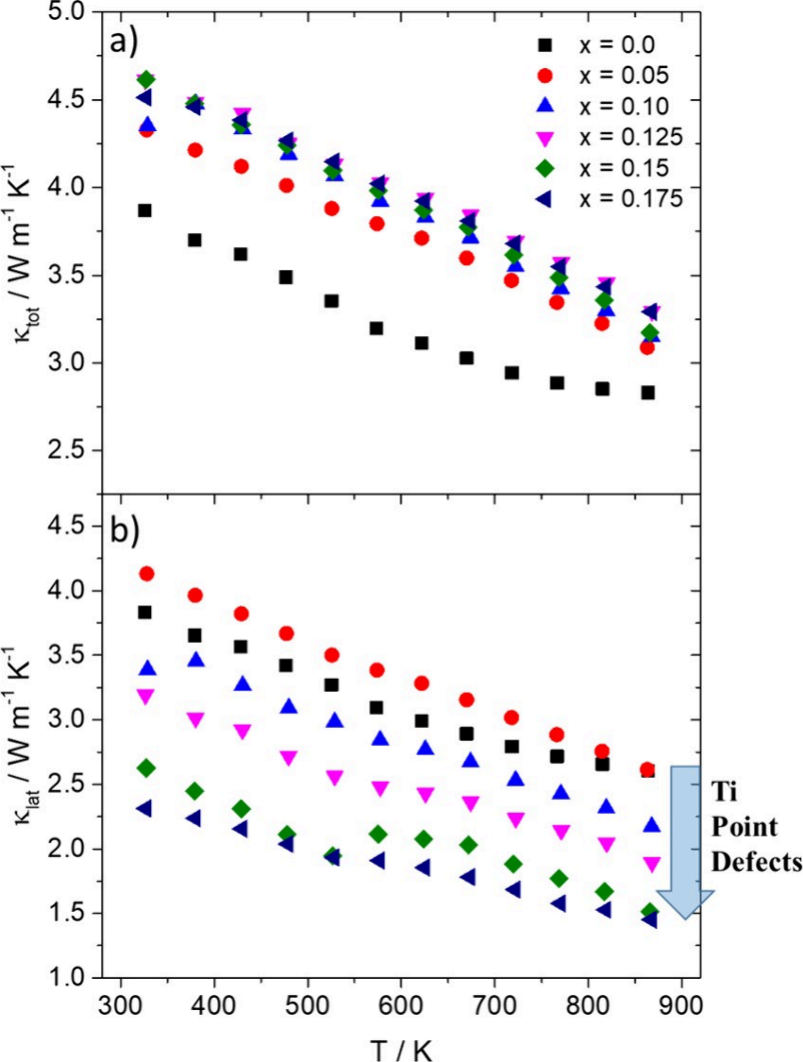
(a) Total and (b) lattice thermal conductivities as a function of temperature for the Nb_0.6_−_ *x*_Ti_*x*_Ta_0.4_FeSb (0 ≤ *x* ≤ 0.175) series.

It is remarkable that the most heavily doped phases with *x* ≥ 0.15 exhibit an appreciable decrease in lattice thermal conductivity across the whole temperature range, reaching *ca*. 35% close to room temperature. As discussed later, the introduction of lighter Ti atoms in the lattice of the (Nb,Ta)­FeSb solid solution results in large mass fluctuations, causing effective phonon scattering at the atomic scale. Comparing the κ_lat_ data of this work with those of previous studies on (Ti,Nb)­FeSb,^48,51,69^ Fe­(V_0.6_Nb_0.4_)_1−*x*_Ti_*x*_Sb,^43^ (Hf,Nb)­FeSb, or (Zr,Nb)­FeSb systems,^42^ it is concluded that the combination of Ti (*m*_a_ = 47.87*u*), Nb (*m*_a_ = 92.91*u*), and Ta (*m*_a_ = 180.95*u*) atoms at the 4*a* site of the HH structure induces a stronger phonon scattering due to the point defects of Ti atoms as well as the Ta/Nb alloying of the system. In Figure S9, the κ values of the pristine phase, Nb_0.6_Ta_0.4_FeSb, are compared with those obtained for the end-member phases, NbFeSb and TaFeSb, from Zhu et al.'s study.^52^ Taking into account that all non-doped phases present poor electronic properties, we can conclude that Nb/Ta alloying causes a powerful phonon scattering in the lattice, markedly reducing the thermal conductivity of the system. The introduction of Ti point defects increases further the complexity of the lattice, and as a result, the Nb_0.425_Ti_0.175_Ta_0.4_FeSb phase exhibits an impressively low κ_lat_ of 2.3 W m^−^^1^ K^−1^ at room temperature, which is one of the lowest reported values along with those of Ta_0.74_V_0.1_Ti_0.16_FeSb,^52^ Nb_0.48_Ta_0.32_Ti_0.2_FeSb,^53^ Ta_0.47_Nb_0.3_V_0.1_Ti_0.13_FeSb,^54^ and Nb_0.25_Ta_0.25_Ti_0.25_V_0.25_FeSb^55^ phases. Possibly, the nanostructuring effect which comes from the ball milling process of prepared materials is another factor that contributes to the overall reduction of lattice thermal conductivity in the Nb_0.6−*x*_Ti_*x*_Ta_0.4_FeSb series. TEM analysis strongly supports this evidence, since it showed a low average particle size of 172 nm (lower than that of Zhu et al.^52^), along with a high nanograin distribution. As a result, a large number of grain boundaries are created in the microstructure of prepared phases that cause a favorable scattering of phonons with a medium mean free path.

The above conclusions, based on the analysis of the experimental data, are supported by the results of *ab initio* calculations of the lattice part of the thermal conductivity. Those are compared to the experimental measurements in the following. Nevertheless, for the compounds considered here, the electronic contribution to the thermal conductivity is significant. As a result, estimating the lattice thermal conductivity using the Wiedemann−Franz law without explicitly accounting for the electronic band structure can only provide an approximate value, and therefore the agreement between the *ab initio* calculations and the experimental values cannot be perfect. This approach remains useful however for gaining insight into the mechanisms responsible for the reduction of lattice thermal conductivity induced by Ti substitution in Nb_0.6_Ta_0.4_FeSb.

Figure 9a presents *ab initio* calculations of the lattice thermal conductivity for several Ti concentrations. Despite the aforementioned approximation, the agreement between the theory and experiment is excellent. The calculations show that the lattice thermal conductivity decreases with an increasing Ti content. At first glance, this trend may appear counterintuitive: increasing the Ti content reduces the average atomic mass, which should enhance phonon group velocities. This effect is indeed observed in Figure 10c, for long-wavelength, heat-carrying phonons.

**Figure 9:**
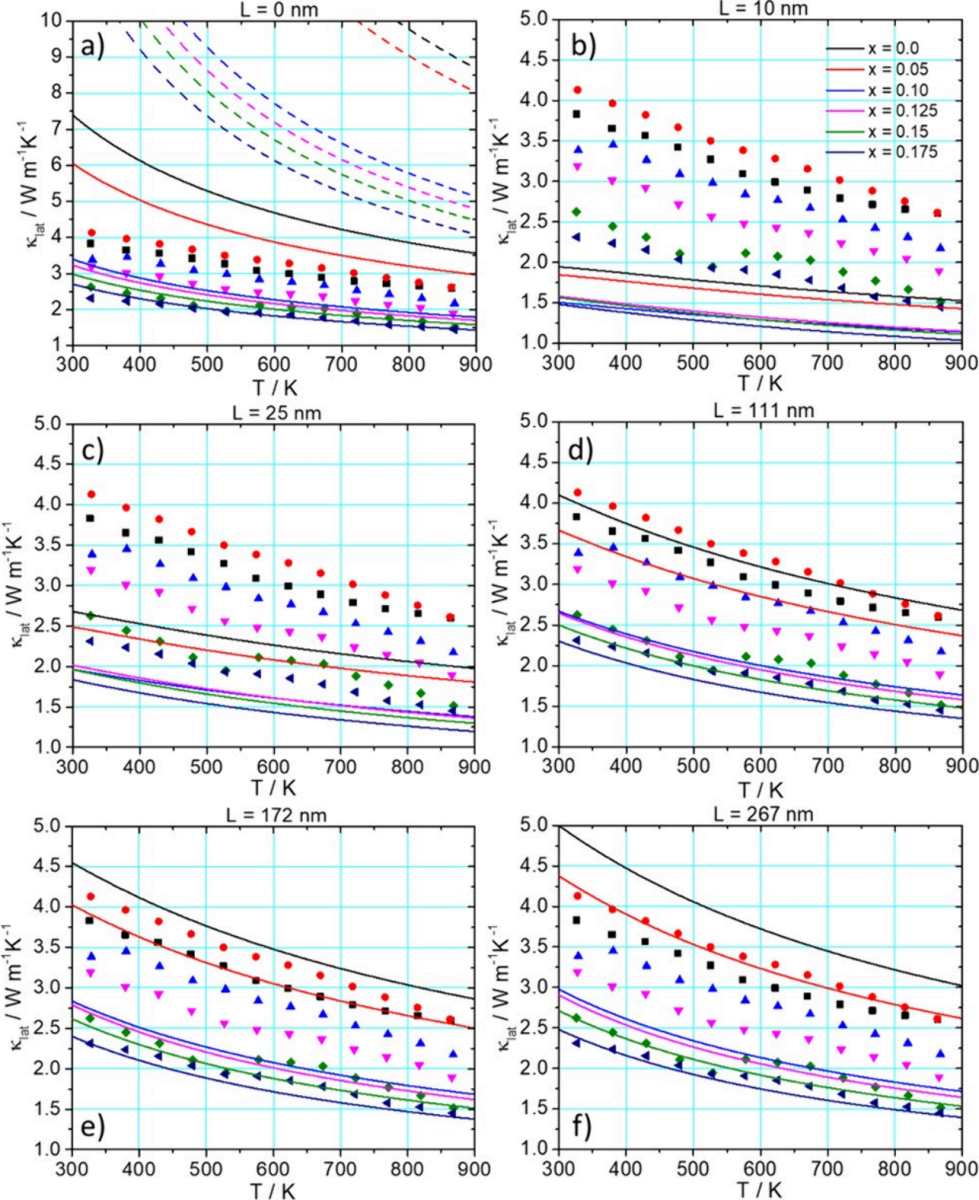
Calculated lattice thermal conductivities (continuous lines), in comparison with those obtained by the experimental measurements (symbols) for different nanoparticle sizes: (a) *L* = 0, (b) 10, (c) 25, (d) 111, (e) 172, and (f) 267 nm. To obtain the *L* = 0 value, numerical computations are performed using *L* = 1 m. The continuous lines are the results of the calculation. For the *L* = 0 case, the dashed lines show the results of the calculations when only phonon−phonon scattering is considered, evidencing the effectiveness of the mass fluctuation scattering.

**Figure 10:**
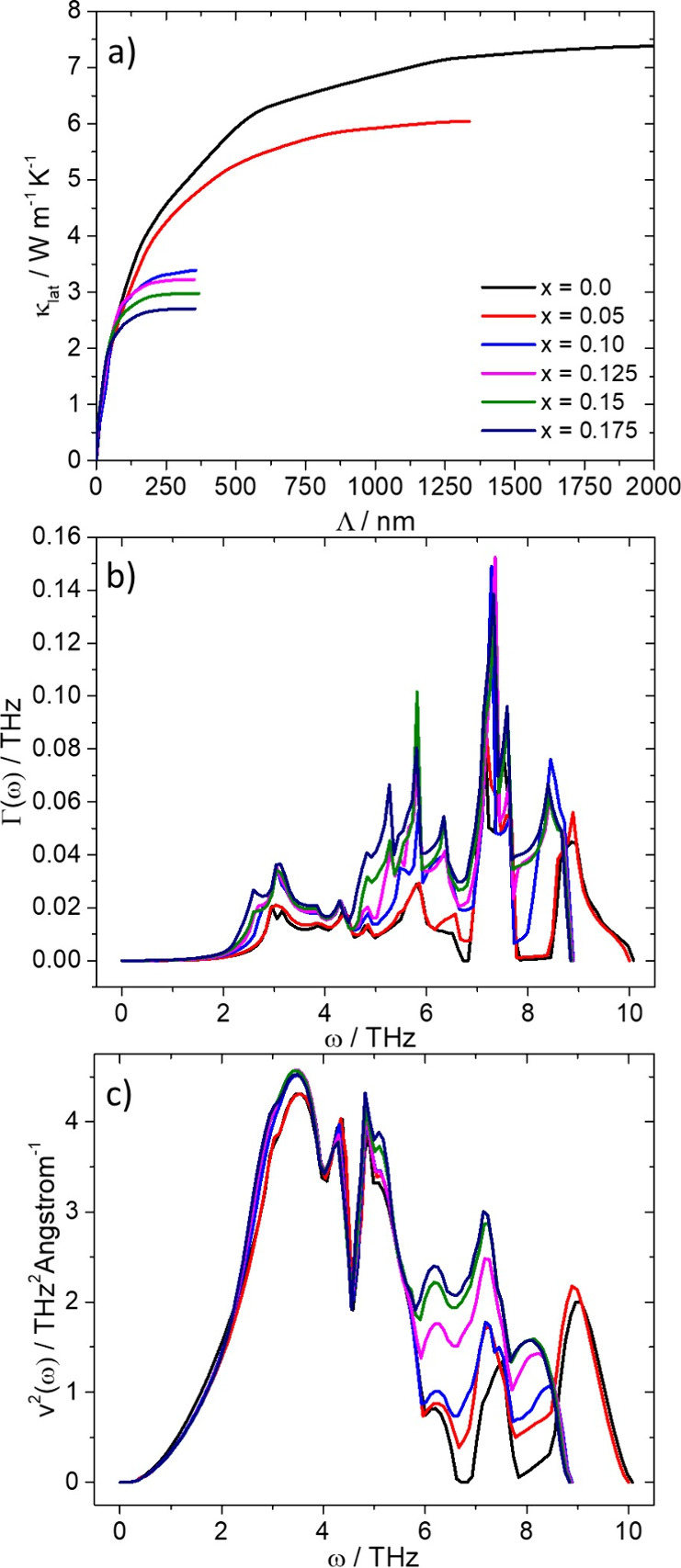
(a) Cumulative lattice thermal conductivities at *T* = 300 K, as a function of the phonon mean free path Λ, (b) imaginary part of phonon−phonon self-energy as a function of phonon frequency, and (c) group velocity squared as a function of the phonon frequency.

However, as shown in Figure 10b, increasing Ti content also enhances the phonon−phonon self-energy for these long-wavelength phonons, leading to a reduction in their relaxation times. In addition, mass fluctuation increases with Ti content, further reducing the total phonon relaxation time entering the Boltzmann transport equation. Consequently, the observed decrease in lattice thermal conductivity with increasing Ti concentration arises primarily from reduced phonon lifetimes, driven by both stronger phonon−phonon interactions and enhanced mass fluctuation scattering. In Figure 9a, the continuous lines show the results of the computations when the relaxation time includes both phonon−phonon interactions and mass fluctuation scattering, while the dashed lines are the result of the computations considering only the phonon−phonon interaction. It is clear from those results that mass fluctuation scattering is indeed very strong.

Figure 9b−f show calculated lattice thermal conductivities that include an additional scattering mechanism: phonon scattering at nanoparticle boundaries. Several nanoparticle sizes were considered (*L* = 10, 25, 111, 172, and 267 nm). The best agreement with experimental data is obtained for *L* = 111 and 172 nm. In contrast, scattering from smaller nanoparticles (10−25 nm) appears too strong to be consistent with the measured thermal conductivities. This suggests that the effective concentration of small nanoparticles is relatively low compared to larger ones, in agreement with the experimental observations of TEM.

The cumulative lattice thermal conductivity of the bulk compounds (*L* = 0) at 300 K is shown in Figure 10a as a function of phonon mean free path. For heavily Ti-doped compounds (*x* ≥ 0.1), most heat-carrying phonons have mean free paths below 100 nm. At lower Ti concentrations, phonons with significantly longer mean free paths contribute to thermal transport. This behavior reflects the increased scattering rates at higher Ti content: phonons travel shorter distances before being scattered by mass disorder or phonon−phonon interactions. As a result, larger nanoparticles (with sizes above 100 nm) are more effective at scattering phonons in compounds with low Ti content, where longer mean free paths are still relevant. In contrast, at higher Ti concentrations, phonons are already strongly scattered before traveling such distances, reducing the relative impact of nanostructuring. In this regime, nanostructuring acts as an additional, but less dominant, scattering mechanism. This trend is consistent with Figure 9, where a strong reduction in lattice thermal conductivity for *x* ≥ 0.1 is only observed for the smallest nanoparticle sizes (*L* = 10 and 25 nm).

Figure 11 presents the TE figure of merit, *ZT*, of Nb_0.6−*x*_Ti_*x*_Ta_0.4_FeSb (0 ≤ *x* ≤ 0.175) series, as determined from the aforementioned physical property measurements. There is a significant improvement of *ZT* with the increase of Ti content analogous with that of PF, and the competition for the best TE performance includes the heavily doped phases with *x* ≥ 10% Ti. Among them, Nb_0.45_Ti_0.15_Ta_0.4_FeSb exhibits the highest efficiencies across the whole temperature range. Reaching a *ZT* of 1 at the temperature of 600 °C, Nb_0.45_Ti_0.15_Ta_0.4_FeSb has proved to be a quite promising candidate TE material for intermediate temperature applications, operating efficiently even closer to the temperature range where most industrial processes take place (400−600 °C).^48^ In addition, previous studies have shown an excellent thermal stability for the NbFeSb-based phases up to the temperature of 873 K.^22^ Repeatability validation of thermoelectric performance was carried out for a representative Ti-doped sample which was synthesized and fabricated two times. Two samples of the stoichiometry Nb_0.50_Ti_0.10_Ta_0.4_FeSb were developed by implementing the synthesis and hot-pressing conditions described previously. The TE figure-of-merit *ZT* of two samples is presented in Figure S10, showing a very good repeatability of the TE performance across the whole temperature range. Although the solubility of Ti can be extended at higher contents, as previous studies have shown,^48,51^ the optimum hole doping for tuning the TE properties and maximizing the performance in (Nb,Ta)­FeSb is accomplished for *x* = 0.15. This doping level (*x* = 0.15) is quite close to the optimum Ti content of previous studies,^52,54^ enhancing the validity of our results. Comparing the performance of our system with those of two end-member phases at 600 °C, the Ti-doped TaFeSb exhibits a higher *ZT*, due to the enhanced PF values. However, Nb/Ta alloying is favorable for the reduction of lattice thermal conductivity. On the other hand, comparing the performance of Nb_0.45_Ti_0.15_Ta_0.4_FeSb with those of Nb_1−*x*_Ti_*x*_FeSb phases at 600 °C,^48,51^ it is shown that Nb/Ta alloying results in a higher efficiency than Nb end-member phases with an optimum Ti doping lower than *x* = 0.2. In addition, Nb_0.45_Ti_0.15_Ta_0.4_FeSb is proven to be more beneficial than other optimized Ti-doped systems such as (Nb,V)­FeSb^43^ and (Hf,Zr)­CoSb_0.8_Sn_0.2_,^72^ exhibiting an improved performance at intermediate temperatures as well as a lower cost. Compared with other HH phases,^42,52,54,73,74^ this compound provides a cheaper solution for large-scale applications, avoiding expensive raw materials such as Hf or V and thus reducing the fabrication cost. Moreover, this study proposes a straightforward and energy-efficient synthetic route such as MA, avoiding high-temperature synthesis, while in parallel it allows scaling up of the fabrication process, which is another important criterion for possible commercialization in the future.

**Figure 11:**
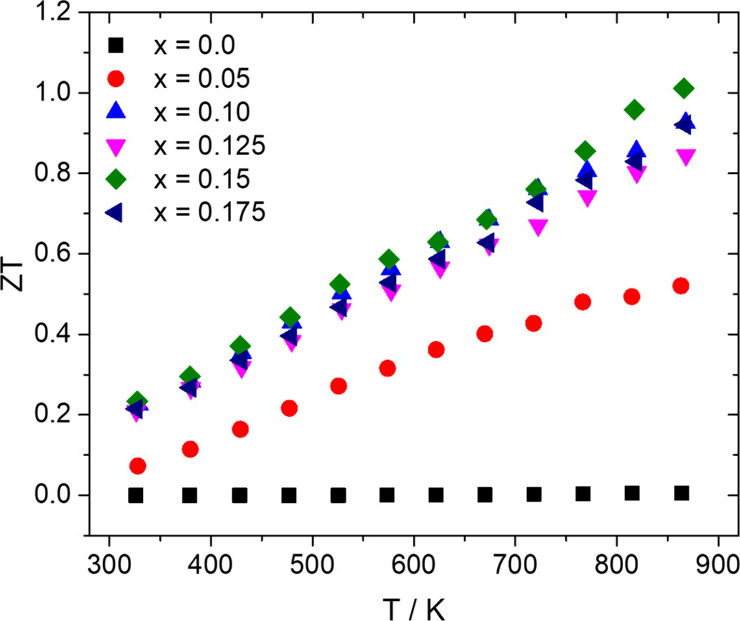
ZT as a function of temperature for the Nb_0.6−*x*_Ti_*x*_Ta_0.4_FeSb (0 ≤ *x* ≤ 0.175) series.

## Conclusions

In conclusion, HH phases of the Nb_0.6−*x*_Ti_*x*_Ta_0.4_FeSb (0 ≤ *x* ≤ 0.175) series were prepared by MA and hot-press sintering. Powder XRD and TEM analyses validate the crystal structure of HH phases. Ti substitution causes an effective hole doping, resulting in a remarkable increase in electrical conductivity, well supported by the evolution of total and site-decomposed electronic DOS near *E*_F_, as determined by the KKR-CPA method. However, the Seebeck coefficient does not show an analogous reduction with the increase in hole concentration, especially for the Ti-rich phases with *x* > 0.10. Electronic structure calculations reveal that Ti, due to strong DOS variations, seems to contribute to the enhancement of sharpness of DOS close to the Fermi level. Theoretical values of the Seebeck coefficient, estimated from the simplified Mott formula at 400 K, are in fair agreement with experimental data. Moreover, the increase of the structural complexity by combining Ti substitution and Nb/Ta alloying, along with the nanostructuring effect of the ball milling process, causes effective phonon scattering, resulting in an ultralow lattice thermal conductivity. Theoretical calculations of lattice thermal conductivity support quite well the experimental results, deciphering the phonon scattering mechanisms in the lattice of investigated materials. The important role of Ti in the reduction of phonon lifetime is revealed due to the strong increase of mass fluctuation scattering. The Nb_0.45_Ti_0.15_Ta_0.4_FeSb phase exhibits the best TE performance, reaching the maximum *ZT* of 1 at 600 °C, and highlights the role of Ti in the TE efficiency improvement of (Nb,Ta)­FeSb phases.

## Supplementary Material



## Notes

Powder XRD Rietveld refinement profiles for Nb_0.6−*x*_Ti_*x*_Ta_0.4_FeSb (0 ≤ *x* ≤ 0.175), total energy versus lattice parameter with determined minimum values for Nb_0.6−*x*_Ti_*x*_Ta_0.4_FeSb computed by the KKR-CPA method, variation of total and site-decomposed (per atom) KKR-CPA DOS at *E*_F_ in Nb_0.6−*x*_Ti_*x*_Ta_0.4_FeSb versus Ti content, thermal conductivity of pristine Nb_0.6_Ta_0.4_FeSb compared to the end-member phases, TaFesb and NbFeSb, repeatability performance test for a representative sample, and the experimental densities of fabricated pellets and their theoretical values, calculated by Rietveld analysis (PDF)
