# Supplementary material for: The Role of Ti in the Improvement of Thermoelectric Performance in Nb0.6−x Ti x Ta0.4FeSb
Source: Chem Mater. 2026 Jun 20;38(13):6347–59. doi: 10.1021/acs.chemmater.6c00307 (PMC13374732; doi:10.1021/acs.chemmater.6c00307)
Supplement: Supplementary file 1 [file cm6c00307_si_001.pdf]

The role of Ti in the improvement of thermoelectric performance in $Nb_{0.6-x}Ti_xTa_{0.4}FeSb$

Panagiotis Mangelis,[a*] *Michal Rybski,*[b] *Mingming Guo,*[c] *Panagiotis S. Ioannou,*[a] *Savvas Hadjipanteli,*[a] *Panagiotis Koutsogiannis,*[d] *Ioannis Thanoglou,*[e] *Andreas Delimitis,*[e] *Laurent Chaput,*[c] *Janusz Tobola,*[b] *Theodora Kyratsi* [a*]

[a]Department of Mechanical and Manufacturing Engineering, University of Cyprus, Nicosia, 2109, Cyprus

[b]Faculty of Physics and Applied Computer Science, AGH University, Krakow, 30-059, Poland

[c]Université de Lorraine, CNRS, LEMTA, Nancy, F-54000, France

[d]Electron Microscopy Core Facility, University of Cyprus, Nicosia, 2109, Cyprus

[e]Department of Physics, Aristotle University of Thessaloniki, Thessaloniki, 54124, Greece

[*] mangelis.panagiotis@ucy.ac.cy, kyratsi.theodora@ucy.ac.cy

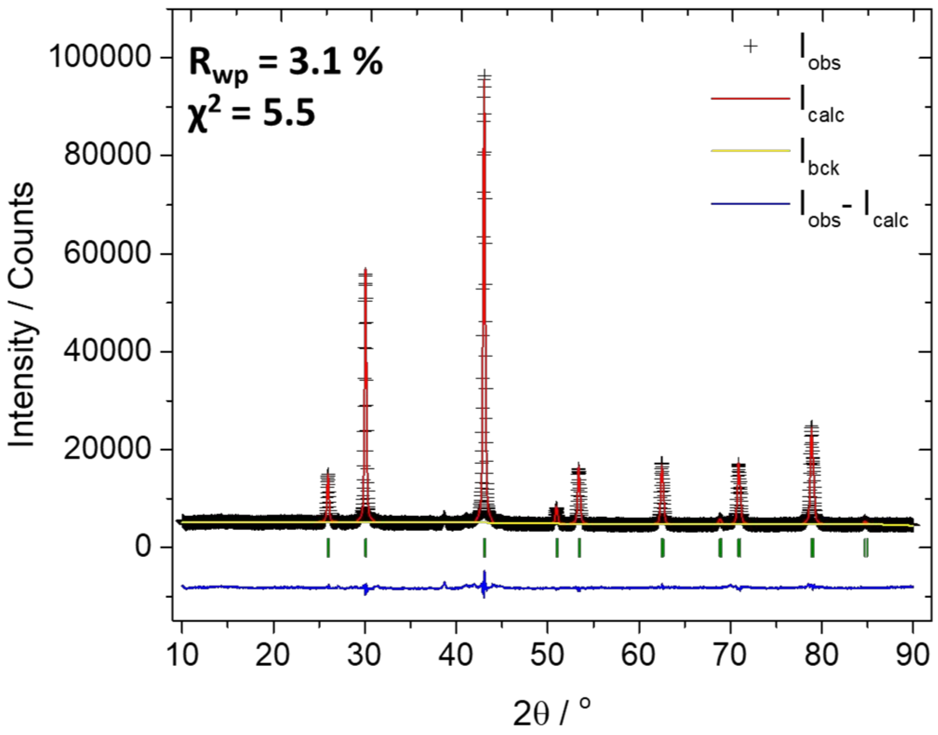

Figure S1 Powder XRD Rietveld refinement profile for the $Nb_{0.6}Ta_{0.4}FeSb$ phase: final observed (black crosses), calculated (red solid line), calculated background (yellow line) and difference (blue line). Reflection positions for the half Heusler phase are marked with olive colour.

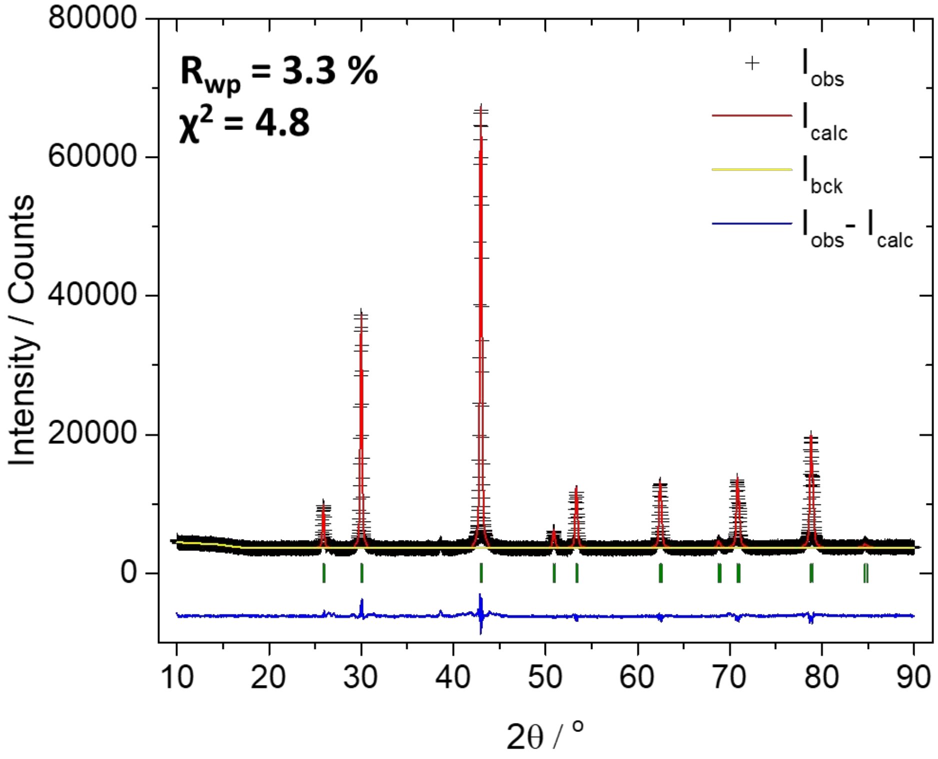

Figure S2 Powder XRD Rietveld refinement profile for the $Nb_{0.55}Ti_{0.05}Ta_{0.4}FeSb$ phase: final observed (black crosses), calculated (red solid line), calculated background (yellow line) and difference (blue line). Reflection positions for the half Heusler phase are marked with olive colour.

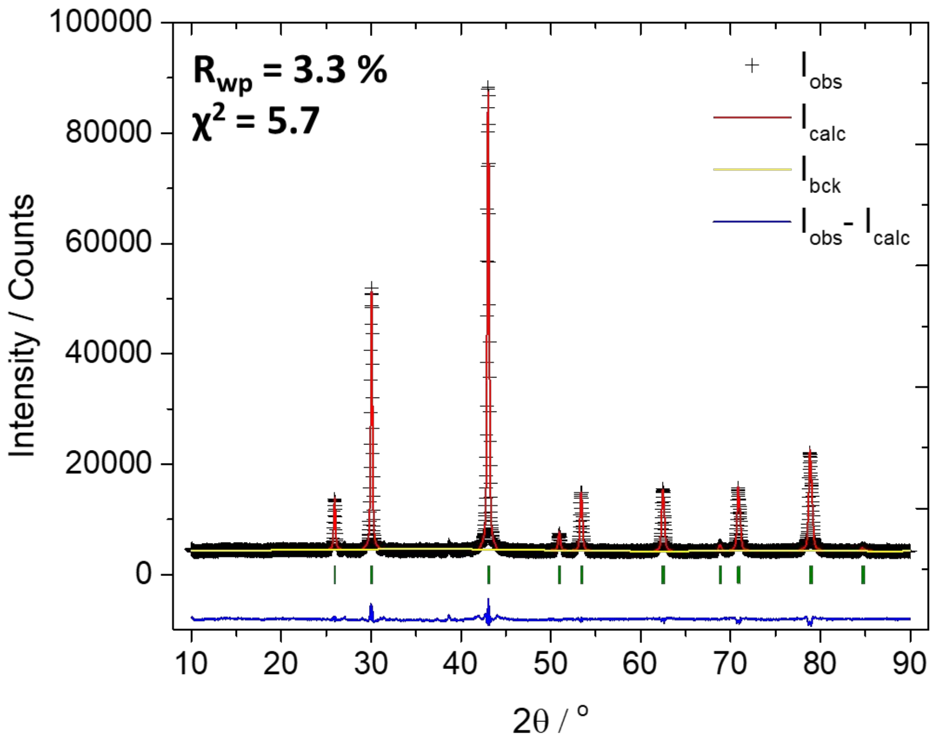

Figure S3 Powder XRD Rietveld refinement profile for the $Nb_{0.5}Ti_{0.10}Ta_{0.4}FeSb$ phase: final observed (black crosses), calculated (red solid line), calculated background (yellow line) and difference (blue line). Reflection positions for the half Heusler phase are marked with olive colour.

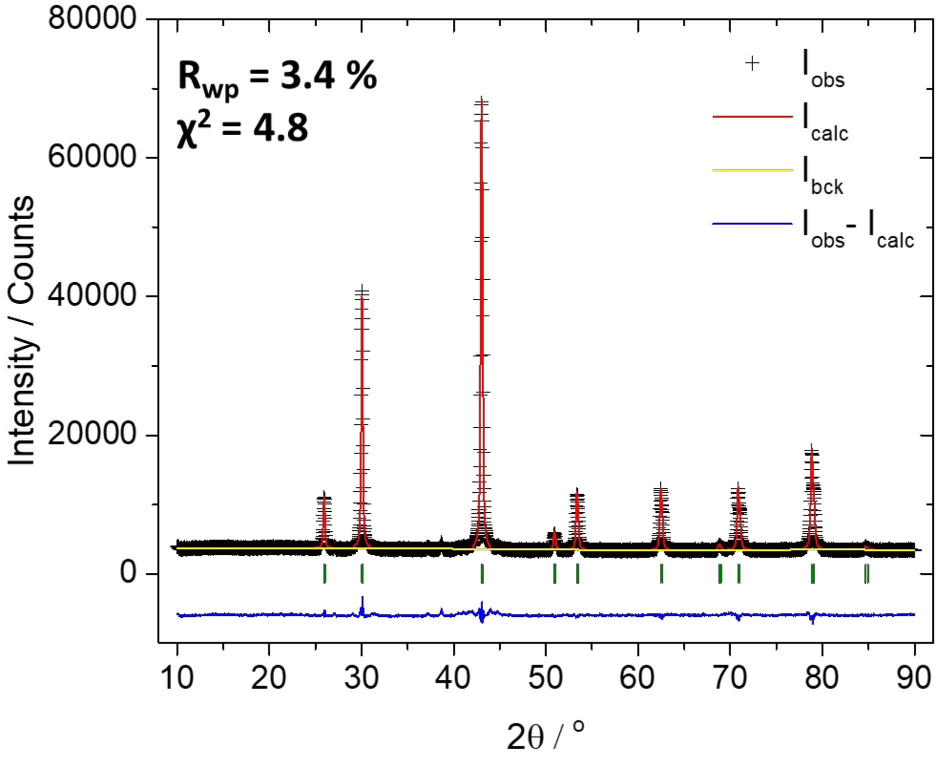

Figure S4 Powder XRD Rietveld refinement profile for the $Nb_{0.475}Ti_{0.125}Ta_{0.4}FeSb$ phase: final observed (black crosses), calculated (red solid line), calculated background (yellow line) and difference (blue line). Reflection positions for the half Heusler phase are marked with olive colour.

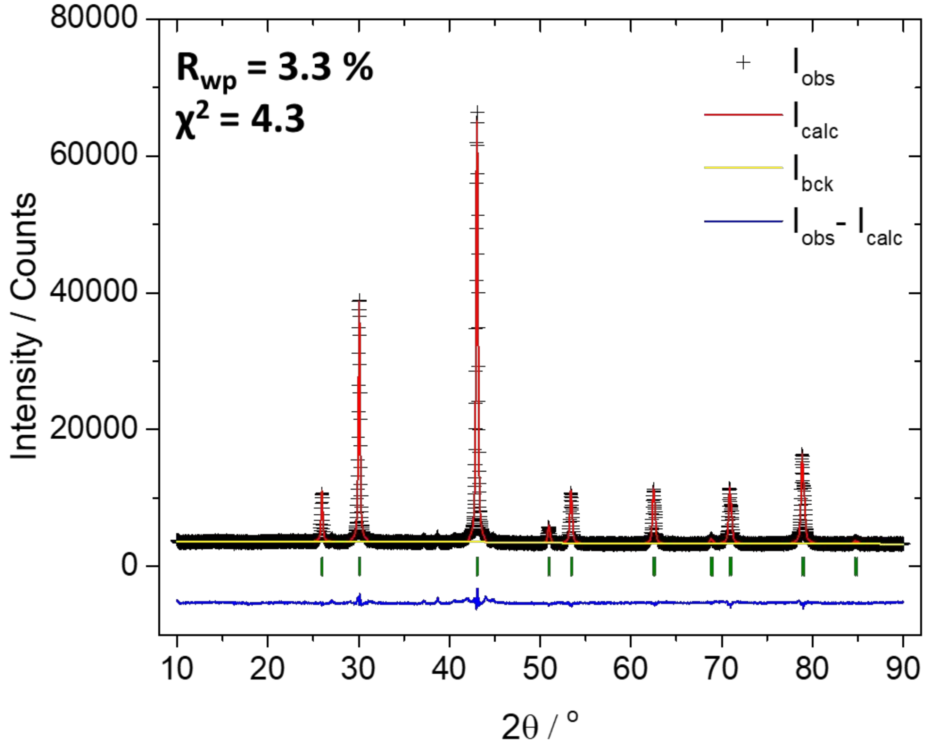

Figure S5 Powder XRD Rietveld refinement profile for the $Nb_{0.425}Ti_{0.175}Ta_{0.4}FeSb$ phase: final observed (black crosses), calculated (red solid line), calculated background (yellow line) and difference (blue line). Reflection positions for the half Heusler phase are marked with olive colour.

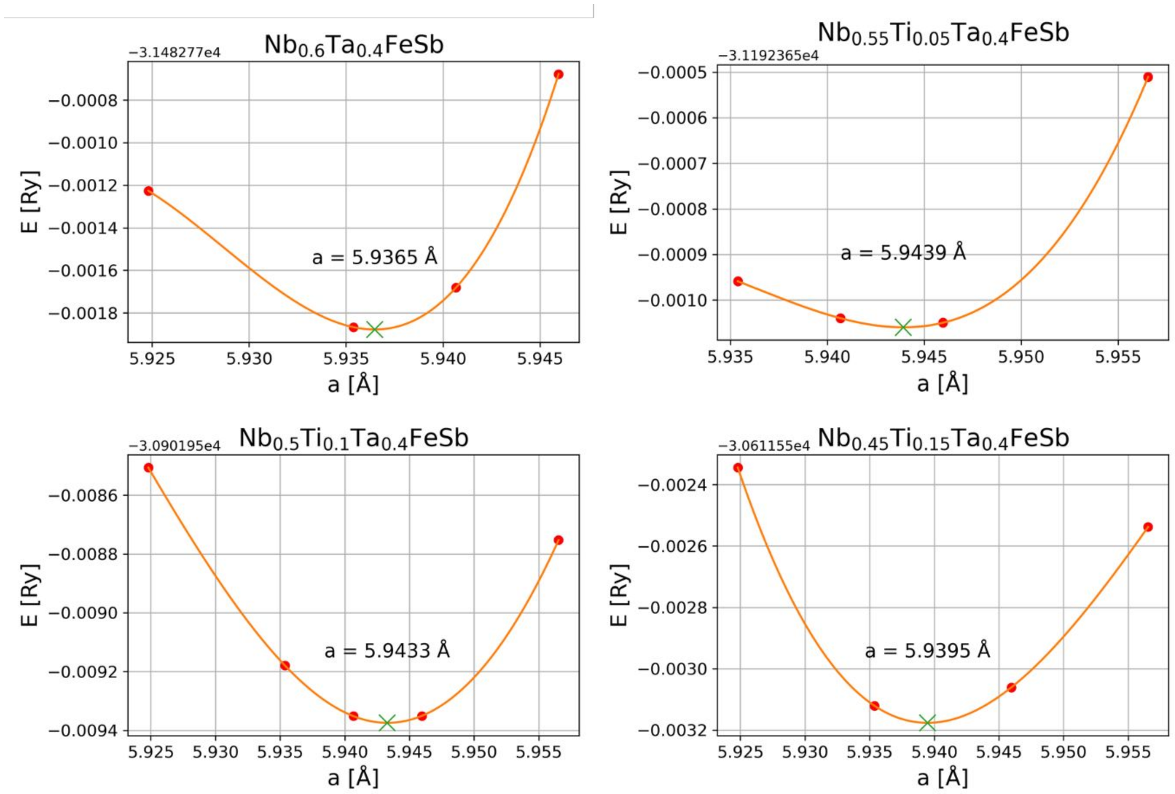

Figure S6 Total energy versus lattice parameter in $Nb_{0.6-x}Ti_xTa_{0.4}FeSb$ for x=0, 0.05, 0.10, 0.15 computed by the KKR-CPA method with determined minimum values.

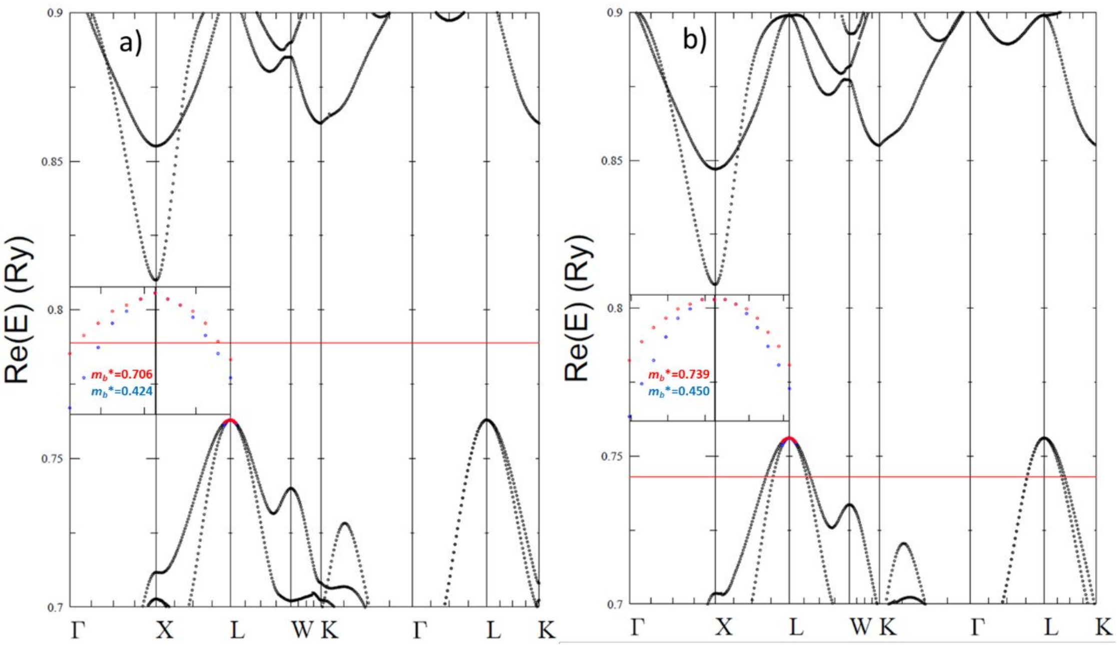

Figure S7 Electronic band structure calculations using the KKR-CPA method for a) $Nb_{0.6}Ta_{0.4}FeSb$ and b) $Nb_{0.45}Ti_{0.15}Ta_{0.4}FeSb$ phases, exhibiting a VBM at L point. Insets: Evaluation of band curvature at the VBM and estimation of band effective mass m_b* using a parabolic approximation.

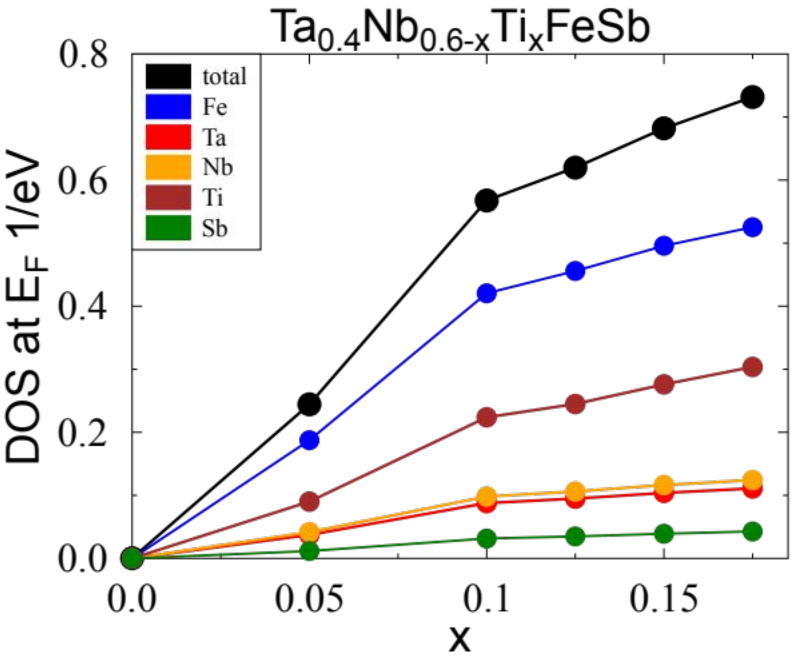

Figure S8 Variation of total and site-decomposed (per atom) KKR-CPA DOS at E_F in $Nb_{0.6-x}Ti_xTa_{0.4}FeSb$ with Ti content.

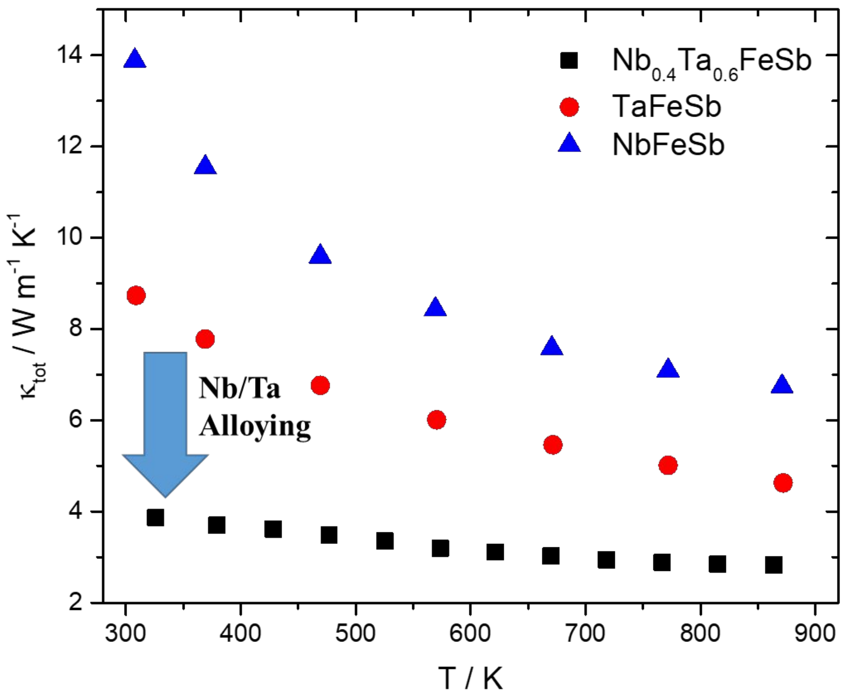

Figure S9 Thermal conductivity of pristine $Nb_{0.6}Ta_{0.4}FeSb$ in comparison with those of non-doped end-member phases, TaFesb and NbFeSb, derived by Zhu *et al*. study.[50]

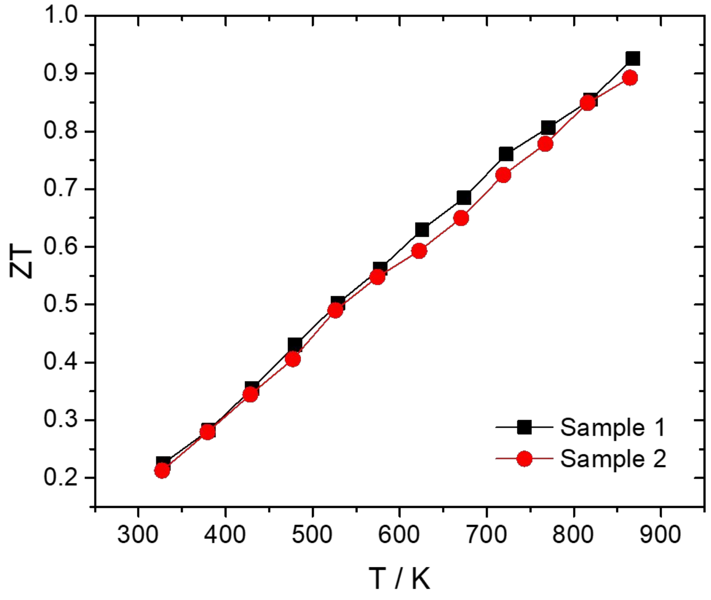

Figure S10 Repeatability performance test in two different samples of stoichiometry $Nb_{0.5}Ti_{0.1}Ta_{0.4}FeSb$.

Table S1 Densities of fabricated pellets in the series $Nb_{0.6-x}Ti_xTa_{0.4}FeSb$ ($0 \leq x \leq 0.175$): experimental and calculated values based on Rietveld analysis.

x	Experimental / g cm^{-3}	Calculated / g cm^{-3}
0	9.45	9.672
0.05	9.38	9.604
0.10	9.35	9.528
0.125	9.29	9.497
0.15	9.23	9.497
0.175	9.23	9.426
